# A Model of Lipid-Free Apolipoprotein A-I Revealed by Iterative Molecular Dynamics Simulation

**DOI:** 10.1371/journal.pone.0120233

**Published:** 2015-03-20

**Authors:** Xing Zhang, Dongsheng Lei, Lei Zhang, Matthew Rames, Shengli Zhang

**Affiliations:** 1 Department of Applied Physics, Xi'an Jiaotong University, Xi'an, Shaanxi, People's Republic of China; 2 Molecular Foundry, Materials Sciences Division, Lawrence Berkeley National Laboratory, Berkeley, California, United States of America; Russian Academy of Sciences, Institute for Biological Instrumentation, RUSSIAN FEDERATION

## Abstract

Apolipoprotein A-I (apo A-I), the major protein component of high-density lipoprotein, has been proven inversely correlated to cardiovascular risk in past decades. The lipid-free state of apo A-I is the initial stage which binds to lipids forming high-density lipoprotein. Molecular models of lipid-free apo A-I have been reported by methods like X-ray crystallography and chemical cross-linking/mass spectrometry (CCL/MS). Through structural analysis we found that those current models had limited consistency with other experimental results, such as those from hydrogen exchange with mass spectrometry. Through molecular dynamics simulations, we also found those models could not reach a stable equilibrium state. Therefore, by integrating various experimental results, we proposed a new structural model for lipid-free apo A-I, which contains a bundled four-helix N-terminal domain (1–192) that forms a variable hydrophobic groove and a mobile short hairpin C-terminal domain (193–243). This model exhibits an equilibrium state through molecular dynamics simulation and is consistent with most of the experimental results known from CCL/MS on lysine pairs, fluorescence resonance energy transfer and hydrogen exchange. This solution-state lipid-free apo A-I model may elucidate the possible conformational transitions of apo A-I binding with lipids in high-density lipoprotein formation.

## Introduction

High-density lipoprotein (HDL) has been intensively studied for decades due to its inverse correlation with the risk of cardiovascular diseases (CVDs) [1]. Through reverse cholesterol transport (RCT), HDL promotes cholesterol efflux from tissues to the liver for degradation. The protein component of HDL is apolipoprotein A-I (apoA-I), a 28-kDa 243 residue polypeptide synthesized by the liver and small intestine, which assembles into HDL particles and modulates RCT [2]. In human plasma, apo A-I is found in both the lipid-free and lipid-bound states [3]. The major function of lipid-free apo A-I is to gather efflux cholesterol and other lipid molecules from cells, and congregate them into discoidal HDL by cooperating with the ATP binding cassette transporter A1 (ABCA1), which starts the RCT process [4, 5].

Revealing the structures of biologically active apo A-I in both lipid-free and lipid-bound states are essential in understanding the molecular mechanisms of apo A-I function [6]. Lipid-bound apo A-I has been intensively studied by different experimental strategies [7, 8], such as X-ray crystallography [9, 10], fluorescence resonance energy transfer (FRET) [11, 12], small angle neutron scattering [13, 14] and chemical cross-linking and mass spectrometry [15–17]. Through combining molecular dynamics (MD) simulations with experimental methods researchers have made great progress in refining the three-dimensional structure of apo A-I in lipid-bound states such as discoidal HDL [18–20] and spherical HDL [21, 22]. Some of these methods have also been used to study lipid-free apo A-I [23]. However, determining the solution conformation of lipid-free apo A-I has been met with more challenges, due to its dynamic nature and sensitivity to experimental conditions.

The structure of lipid-free apo A-I was first suggested as a two domain structure containing a helical bundle or an elongated hairpin helix conformation by limited proteolysis and deletion mutants [24, 25]. It was later confirmed by detailed fluorescence studies combined with site-directed mutagenesis [26] and FRET measurements [27]. The helix content of lipid-free apo A-I measured with circular dichroism (CD) spectroscopy indicated that the C and N-terminal domains were independent with helix content in the N-terminus (1–189) at 57±5%, and the total helix content of lipid-free structure in blood plasma at 49±3% [28]. A further study by hydrogen exchange and mass spectrometry (HDX-MS) described the stability of secondary structure [29].

**Fig 1:**
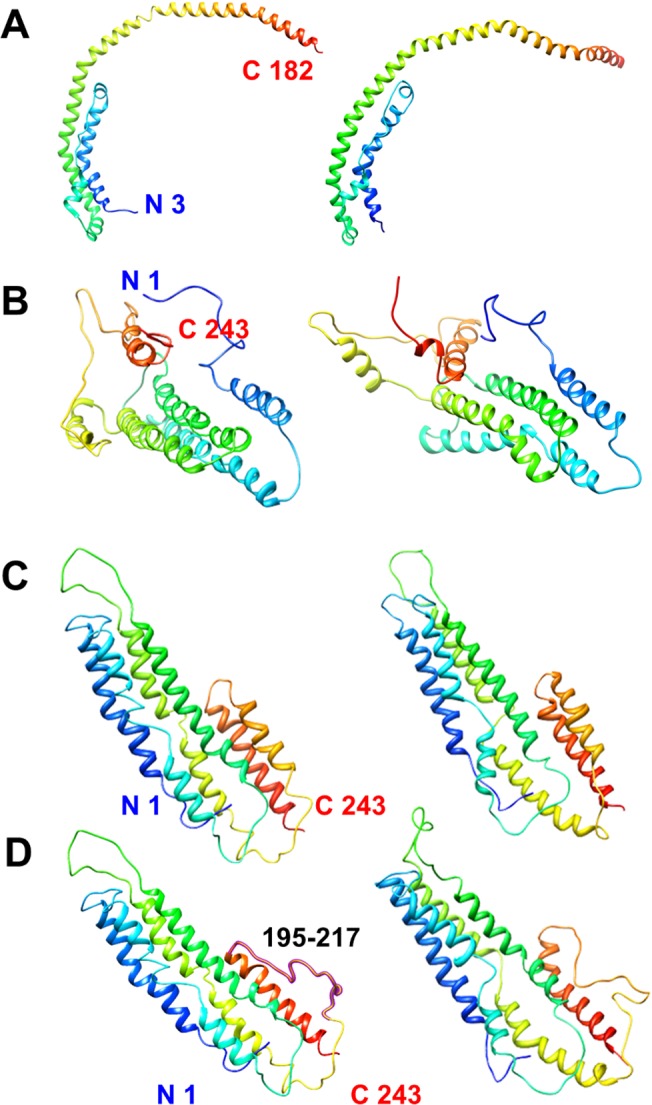
Structural comparison of the four lipid-free models. A) The X-ray model. B) The CCL/MS model. C) The MD model. D) The CMD model. The left panels show the initial structure of each protein while the right panels show structures after simulation. All proteins are represented in ribbons using Chimera. The region of residues 195 to 217 in the CMD model is indicated by purple.

Several models of lipid-free apo A-I have been proposed. One such model was obtained from chemical cross-linking/mass spectrometry (CCL/MS) and homology modeling. By utilizing the Lys distribution information provided by these results, the CCL/MS model described a globular shaped helical cluster, composed of six short helices and loops, exhibiting very close N and C-termini [30] (Fig. 1B left). Another model, called the“beta-clasp” model, has been constructed based on electron paramagnetic resonance spectroscopy (EPR) results and suggests a structure comprised of 51% α-helices and 12% β-strands [31]. Recently, an X-ray crystal structure of the C-terminal truncated apo A-I at 2.2 Å resolution has also been proposed, containing a bent extended α-helical hairpin structure [32] (Fig. 1A left).

Nevertheless, there are some contradictions among these models. First, the three models show different α-helix/β-strand content and distribution. Both the X-ray model and CCL/MS model contain only helices and loops. The CCL/MS model has an α-helix content of 51%, consistent with the CD result [28], and the α-helix distribution shows similarity with both HDX-MS [29] and NMR data [33]. The “beta-clasp” model suggests 12% β-strand, including residues 20–25, 120–129 and 150–158 [31], which deviates greatly from all other results. More importantly, the three models show a divergent tertiary structure: i) the CCL/MS model predicts a more globular shape. ii) the X-ray model exhibits a bent and extended α-helical hairpin shape and iii) the ERS model forms a bundled α-helix and β-strand shape. Two primary aspects of structural determination may cause these differences. First, the distance data of LYS pairs from CCL/MS experiment provides few restrictions on the protein backbone, so various three-dimensional structures are allowed by the CCL/MS result [30]. Secondly, since apo A-I has been shown to exhibit high flexibility [15, 34, 35], experimental processes like protein crystallization for X-ray crystallography will change the conformation of apo A-I under in-vivo conditions [7]. By defining a structure which shows stability under physiological conditions and simultaneously agrees with known experimental results, a unitive structure can be proposed.

In this study, by using MD simulations and comparing to experimental results [36, 37], we tested the current CCL/MS and X-ray models. MD simulation shows that neither of them can achieve an equilibrium state, as indicated by the measurement of root mean-square deviation (RMSD), solvent-accessible surface area (SASA), secondary structural content, and protein potential energy during MD simulations. Furthermore, by using MD simulations along with previous experimental results, we proposed a full length all-atom apo A-I model, molecular dynamics (MD) model, and detailed analysis compared to known experimental results.

## Methods

### Preparation and Energy minimization

Crystal structure of lipid-free apo A-I was taken from protein data bank (PDB entry 3R2P) [32], and the CCL/MS model was obtained from Davidson, W. S. [30]. Before MD simulations, energy minimization was applied to all the initial lipid free apo A-I models with the Cα atoms restrained (force constant of 5 kcal/mol Å^2^), in order to remove steric clashes in the protein structure. The protein was solvated using the VMD solvate plug-in [38] to create a cubic periodic water cell extending 20 Å beyond the protein atoms. Sodium ions were then added to neutralize the system. Additional energy minimization steps were applied to optimize the solvent molecules. The simulation systems had a total of ∼100,000 atoms, including the protein, water molecules and sodium ions.

### Molecular dynamic simulations

Each all-atom MD simulation, including iterative MD simulations for modeling and simulation tests for evaluation, were performed by using the program NAMD2.7b [39]. CHARMM22 force fields [40], including Φ,Ψ cross-term map (CMAP) correction [41], were used for the protein, TIP3P water [42] and ions. Periodic boundary conditions were performed for each system and a cutoff distance for van der Waals interactions was set to 12 Å. The Particle-Mesh Ewald (PME) method [43] was applied to evaluate the electrostatic forces of the systems. The whole system was heated from 0 K to 310 K over 62 ps simulation, using weakly coupled Langevin dynamics [44], and then the temperature was maintained at 310 K. The pressure was maintained constant at 1atm using a Langevin piston Nose-Hoover barostat (with a piston period of 100 fs and a decay time of 50 fs) [45]. A time step of 2fs was used and all bonds to hydrogen atoms were held rigid. All simulations were first carried out with the protein Cα atoms restrained (force constant of 5 kcal/mol Å^2^) for 0.4 ns. The restraints were then removed and the system was allowed to equilibrate freely for 15 ns.

### Construction of MD Model by Iterative MD simulations

A new lipid-free apo A-I molecular structure was proposed based on the known experimental data with an iterative MD simulation method described as below.

First, the secondary structure was modeled referring to the helix conformation of the human Δ1–43 apo A-I crystal structure (PDB entry 1AV1) [9] and the secondary structure indicated by HDX-MS [29] and nuclear magnetic resonance spectroscopy (NMR) experiments [33]. Helices in the lipid-free apo A-I structure were modeled as the 18/5 α-helix. Most Pro and Ala residues were distributed on the edge of the helix or loop based on the helix kink properties shown in the crystal structure of Δ1–43 apo A-I [9]. NMR data of human apo A-I in lipid-mimetic solution was also used as a reference to model the N-terminus of human apo A-I [33].

There is a common prediction that lipid-free apo A-I contains a helix bundle N-terminal domain and a less organized C-terminal domain in which both are composed by α-helices and loops [46–48]. So the secondary structure of the MD model was constructed as 6 helices and 5 loops. Helix 1 is residues 7 to 46，as described in HDX-MS and NMR experiments. Helix 2 is residues 48 to 82 with a small kink in the middle on residue Pro66. Helix 3 is Lys94 to Pro121. Helix 4 is Pro143 to Ala180 with a small loop from Arg160 to Pro165, as suggested by the limited proteolysis experiment [24]. The first four helices in the N-terminal domain constitute a helical bundle. In the C-terminal domain, helix 5 is residues Ala195 to Clu212 and helix 6 is residues from Pro220 to Leu240. Since the experimental results are most varied around the region of helix 5 in the lipid-free apo A-I structure (NMR data suggests it to be helix [33], but X-ray structure showing it to be loop [9]), it was initially modeled as a helix in our MD model for simplicity. The C-terminal domain (residues 192 to 243) was modeled in close proximity to the N-terminal helix bundles as the demonstrated from FRET that the C-termini is nearby Trp residues in the N-termini [48, 49], with hydrophobic surface components oriented to the hydrophobic region on the surface of the N-terminal domain [50]. The atomic coordinates of helical structure were cut from the X-ray structure [9] based on their corresponding residue positions.

Second, the six separate helices of the protein were assembled using VMD, with each helix treated as a rigid body. Side chains obtained from α-helical conformation were kept unchanged from the crystal structure. Different construction patterns of the six helices combinations were modeled in order to exhaust possible conformations (S1 Fig.). The relative positions and orientations between the helices in three-dimensional space were adjusted to minimize the total conformation energy and following criteria was applied to test the results: 1) The Lys on adjacent helices and loops was matched with CCL/MS raw data [30]. 2) The hydrophobic surfaces were reduced in order to lower the surface energy (S2 Fig., hydrophobic analysis was performed with the wheel.pl application created by Don Armstrong and Raphael Zidovetzki. Version: 0.10 p06. 12/14/2001. DLA). 3) Permissible (distance and orientation) salt-bridges and hydrogen bonds [51]. 4) When optional features were allowed, dozens of models were built and examined by the same MD simulation and stability criteria.

Third, the loops (including side chain) were generated using modeling software Modeller9.7 [52], which used a DOPE-based (Discrete Optimized Protein Energy) loop modeling protocol [53]. 5–20 models were generated for each different loop and evaluated by the same evaluation procedure outlined above to search for the best structure. The region near residue 65 was modeled into a short loop as predicted by HDX-MS [29]. The region near residue 174, which was supposed to be a hydrolysis site [24], was modeled into a short loop. After this, we obtained a template model.

Fourth, sequential iterative MD simulations were carried out to find the most probable conformation. Once a template model had been built, energy minimization and 15 ns MD simulation were used to test its stability. Molecular trajectories from the MD simulation and experimental data from all resources were used to guide the iterative construction of the next protein model. Steps two to four were repeated to keep well defined structural portions and replace poorer sections, until a convergent structure was found to agree with most of the experimental data. After 16 rounds, with a total simulation time of over 150ns, a MD model was found to be most reasonable.

Another model was also built, differing from the MD model only in helix 5 to account for HDX-MS experiments, which suggested there is a loop in residues 195–217 [29], called “changed MD” (CMD) model. In the MD model, helix 5 (residue 195–217) was modeled as the NMR data suggested [33], while in the CMD model residues 195–217 were rebuilt as a loop.

### Structural flexibility and activity analysis

We analyzed the entire simulation trajectory obtained after the restraints were removed. Using root mean-square deviation (RMSD), average solvent-accessible surface area (SASA) and potential energy of the protein (using ‘NAMD energy’ plug-in of VMD software) [38], the stability and structural characteristics of each model under the same simulation conditions were evaluated.

Since the MD model exhibited the best structural characteristics by the analysis of MD simulation and multiple short trajectories are more efficient than a single long trajectory for sampling conformational diversity [54], an additional six replicate simulations for this model were carried out in order to check the consistency of results and explore possible neighboring conformational states. The simulation time for this model was 105 ns in total, which ensured the stability estimate and helped to explore conformational fluctuation. The atomic coordinates of MD and CMD model were saved in PDB format (S1 Datasets).

All structural figures were generated with VMD [38] or Chimera [55].

## Results and Discussion

### Structural stability of X-ray, CCL/MS and MD models

The stability of the X-ray and CCL/MS models were tested by energy minimization and MD simulation for 15 ns, and the initial and simulation output models are shown in Fig. 1. Obviously, the conformation of the X-ray model changed after simulation. The CCL/MS model showed reorientation in its secondary structure and redistribution of its tertiary structure. After the simulation all 6 helices moved apart from one another. Half of the helical structure in helix 1 (residue 24 to 39) adopted a loop conformation. Additionally a helix break appeared in helix 2 (residue 48 to 82).

**Fig 2:**
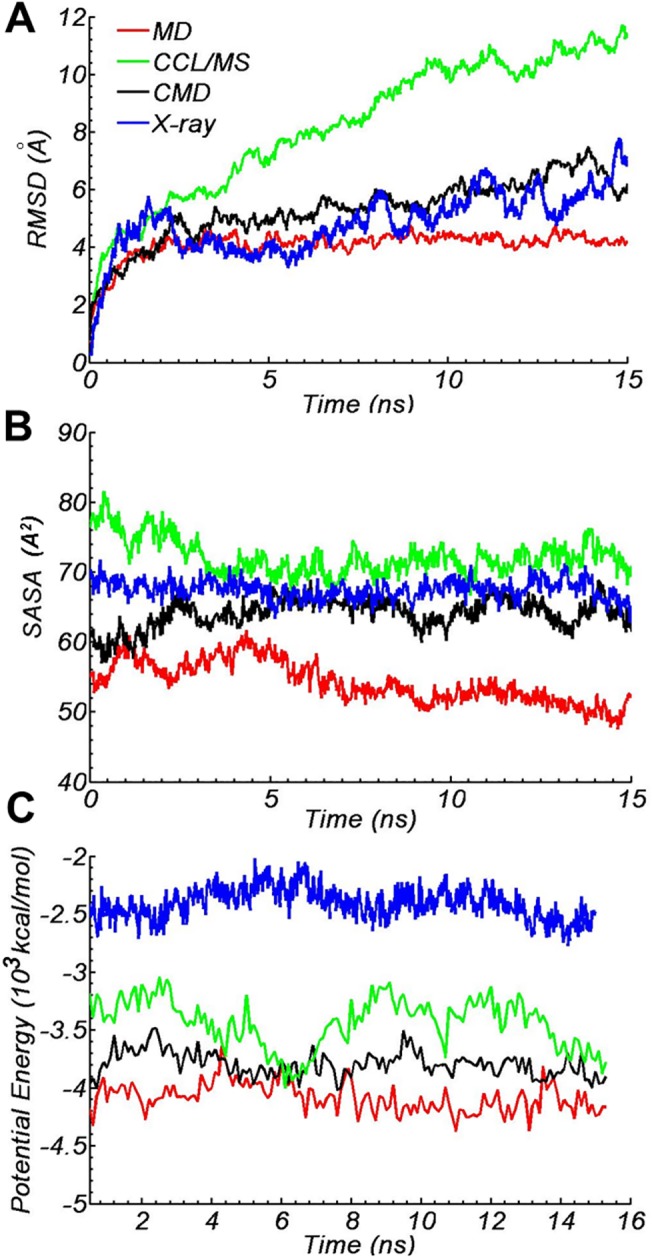
Structural stability of the four models. A) RMSDs of all protein atoms (without H atom) were measured over the entire trajectory. The protein structure in each frame was aligned to the initial structure, respectively. RMSDs were measured every 2 ps during the 15 ns all-atom simulation. B) The average solvent-accessible surface area of every hydrophobic residue exposed to water, measured every 20 ps in the all-atom simulations. SASAs were calculated with a 1.4 Å probe radius and a grid size of 0.25 Å. 8 types of hydrophobic residues were taken into account (Ala Leu Val Ile Pro Phe Met Trp). There are 92 total hydrophobic residues in lipid-free apo A-I models (68 in X-ray model). C) Potential energy comparisons of the four apo A-I models. Potential energy of the protein was measured every 20 ps during the simulations (from 0.4 ns to 15 ns). Potential energy includes bond, angle and dihedral angle energy, electrostatic interactions and Van der Waals interactions. The 0.4ns Cα atoms restrained simulations are not included.

The RMSDs of the proteins in simulation were used to analyze the extent of equilibration that the model had reached. RMSDs of all protein atoms were measured over the entire trajectory after the restraints were removed, shown in Fig. 2A. The RMSDs of the X-ray model and CCL/MS model rose higher than 5 Å within only 4 ns, and neither could reach equilibrium within 15 ns. Relative positions of regions around helices 5 (residue 169 to 186) and 6 (residue 210 to 238) in the CCL/MS model changed significantly after simulation, exhibiting RMSDs of 27.0 Å and 12.2 Å from the initial structure (aligned by helix 2, 3 and 4, residue 48 to 164), respectively.

Since the X-ray model is C-terminal truncated, and both the X-ray model and CCL/MS model are not equilibrated in solution, a new model was necessary to be built to describe solution state of lipid-free apo A-I, which was finally obtained by iterative MD simulations and named the MD model. The CMD model was also built to investigate the local structure in the C-terminal domain, as shown in Fig. 1. The structure after 15 ns simulation remains similar to its initial structure.

In Fig. 2A, the MD model which showed the greatest stability and rapidly reached an equilibrium RMSDs value of 4 Å after 2 ns of simulation with a fluctuation of less than 0.2 Å. CMD model showed less stability and a small fluctuation at 15 ns, which meant a more dynamic conformation induced by the loop of residues 195 to 218 in the C-terminus.

### Comparing the structural stability of the four models

Besides the RMSD, we also used SASA and potential energy of protein (without solution) to evaluate the stability of the four models, CCL/MS, X-ray, MD and CMD model (Fig. 2).

The SASAs of hydrophobic residues can be a physical parameter in evaluating protein structure, especially with lipoproteins. The SASAs data for hydrophobic residues in the CCL/MS model showed a high value of 73 ± 2 Å^2^, higher than those of the X-ray model, 67 ± 2 Å^2^_,_ MD model, 53 ± 2 Å^2^, and CMD model, 64 ± 2 Å^2^ (Fig. 2B). Spacing between helices or loops are generally loose, allowing for water permeation to more exposed protein hydrophobic surfaces within the CCL/MS model. The solution state of apo A-I should be more compact than the CCL/MS model predicts, considering the amphipathic helices of apo A-I [2] and polarized solution environment. A similar more compact structure has also been found in lipid-free apo E [56] and apo A-IV [57].

The potential energy of protein structures can also be used to rate stability. We calculated the potential energy of each model. The MD model showed a lower and flatter energy curve than the others, which corresponds to a more favorable state (Fig. 2C). The energy curves of three other models exhibit bigger fluctuations, indicating the appearance of observable structural alterations, which is also confirmed by RMSDs and SASAs.

Based on these structural stability criteria, the X-ray, CCL/MS and CMD model models show instability while both MD and CMD models exhibited high stability.

### Structural consistency of MD and CMD models to experimental results

**Fig 3:**
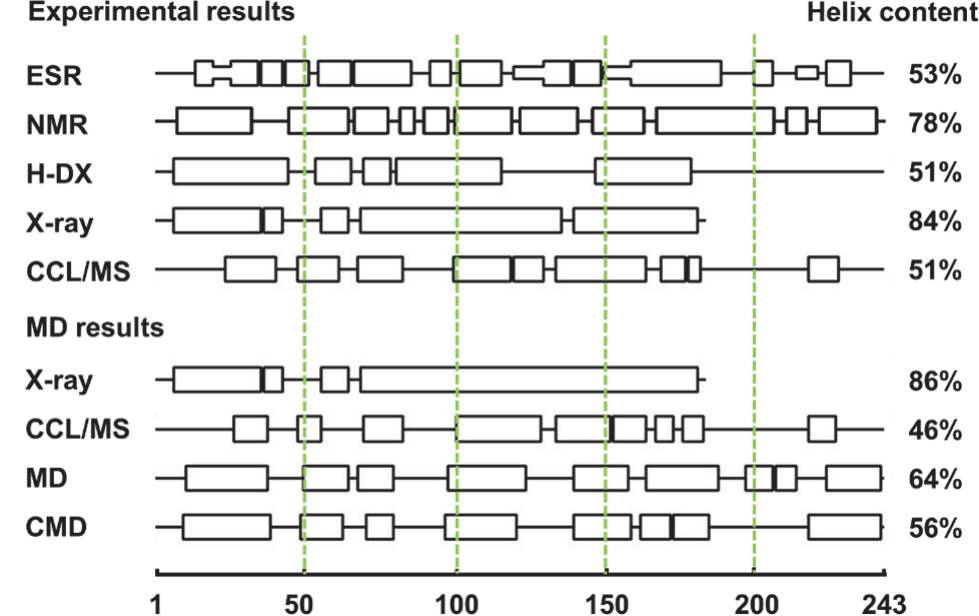
Secondary structure comparisons of experimental lipid-free models and the MD results. The central line represents the residue index. Rectangles along the line represent α-helices. Rectangles with half width represent β-strands. Secondary structures of all models are measured by VMD using a uniform standard. Experimental results are showing on top and structures after MD simulations are show below).

So far, only EPR [31], NMR [33], HDX-MS [29] and X-ray crystallography [32] experiments have provide detailed information for the secondary structure distribution of lipid-free apo A-I. Fig. 3 presents the helix distribution of lipid-free apo A-I from four published experimental data and also the homology CCL/MS model.

In the N-terminal domain of the refined MD model and CMD model, the four-helix conformation was constituted by helix 1 (residues 7–39), helix 2 (50–79, with a short loop on residues 55–57), helix 3 (98–123) and helix 4 (140–186, with a short loop on residues 158–163). During the simulation, the secondary structure of the X-ray model was well conserved (small change around residue 140). In the secondary structure of the N-terminus (residue 1–80), our MD models have a similar (∼75%) secondary structure distribution with the X-ray structure (Fig. 3) [32], yet show a different orientation in helix 3 and helix 4 (residue 85–185) (Fig. 2). While the X-ray model predicts an extended conformation, a bundled helix tertiary structure agrees with previous studies [46–48], and may be the natural folding state in solution.

In the C-terminal domain of MD and CMD models, residues 223 to 238 are helices, as in the EPR experimental data [31] and CCL/MS model [30]. Residues 195 to 218 are a helix in the MD model and a loop in the CMD model, where controversial structures were reported by various experiments. The EPR experiment suggested that a short loop or β-strand is in residues 214 to 220 [31]. The HDX-MS results suggested a helix-free C-terminus [29], while NMR results indicated that these residues form two short helices [33]. In the lipid-bound state of apo A-I this region was suggested to be α-helical and exhibited great hydrophobic properties on the side of the helix [58]. However, the simulation results from the CMD model show that the loop in helix 5 slightly reduced the stability of the whole structure.

**Fig 4:**
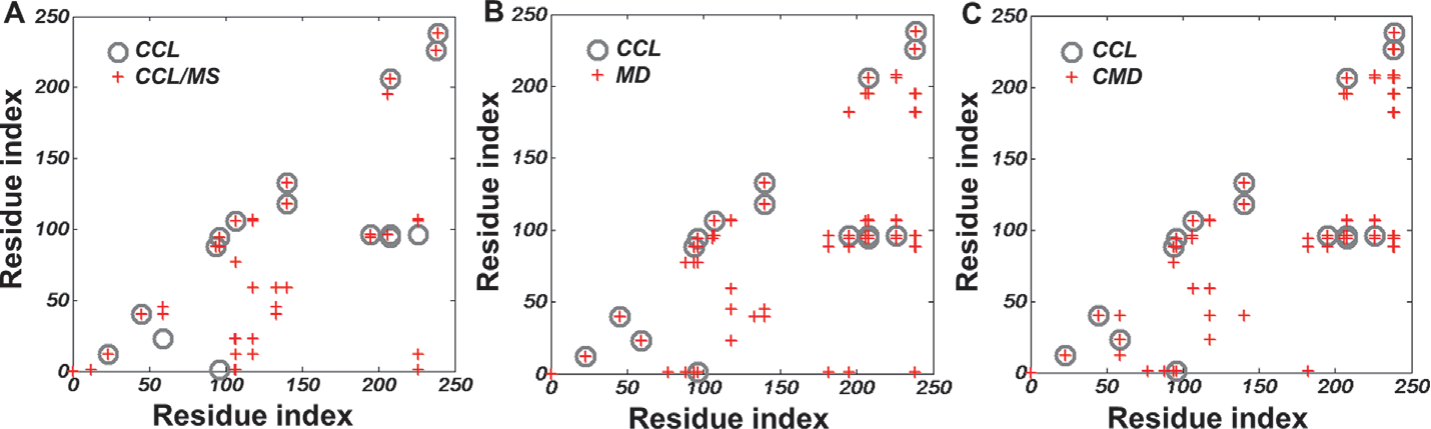
The distribution of neighboring Lys pairs in different lipid-free apo A-I models and their consistency to the CCL/MS experiment. The relative distance of Cβ in Lys residues in the output structure of the simulation is measured by VMD. Hollow circles indicate experimental CCL/MS data (Davison et al [40]). The cut-off distance between two Cβ in Lys residues chosen for the calculation was 20 Å. Gray circles present the experimental data from CCL/MS method. The X and Y axes of the plot indicate the residue number of apo A-I (1–243). The cross linking data is shown through Lys pairs with a CCL link distance of 20 Å (grey circles), and compared to the CCL/MS, MD, and CMD PDB models (red plus signs). Circles with a red plus sign mean the generated model Lys distance agrees with the CCL experimental data, consequently a circle without a plus sign means the CCL data did not match the model.

Chemical cross-linking data imposes a very strong restriction on the tertiary structure of lipid-free apo A-I models. The CCL/MS results indicated 7 inter helix Lys cross-link pairs and 11 intra helix pairs that pose restrictions on the three-dimensional model [30]. Lys pairs within the cutoff distance 20Å were identified in each model before and after simulation, shown in Fig. 4. The X-ray model did not match with the cross-linking data in the N-terminal domain either before or after simulation (distance from residue 23 to 59 is ∼34 Å). Although the CCL/MS model was built from CCL/MS data, during the simulation three interpeptide Lys pairs are out of cross-linking range a majority of the time (Nt1-Lys96, Lys23-Lys59 and Lys96-Lys226, S3 Fig.). Especially the distance between Nt1 and Lys96, as this became larger than 30 Å after 2 ns simulation. On the other hand, in both the MD and CMD models, the Lys pairs remained within the cutoff distance over the entire 15 ns simulation. These results suggest that the CCL/MS model lipid-free apo A-I structure is far from equilibrium and the MD model approximates the solution state of lipid-free apo A-I.

**Fig 5:**
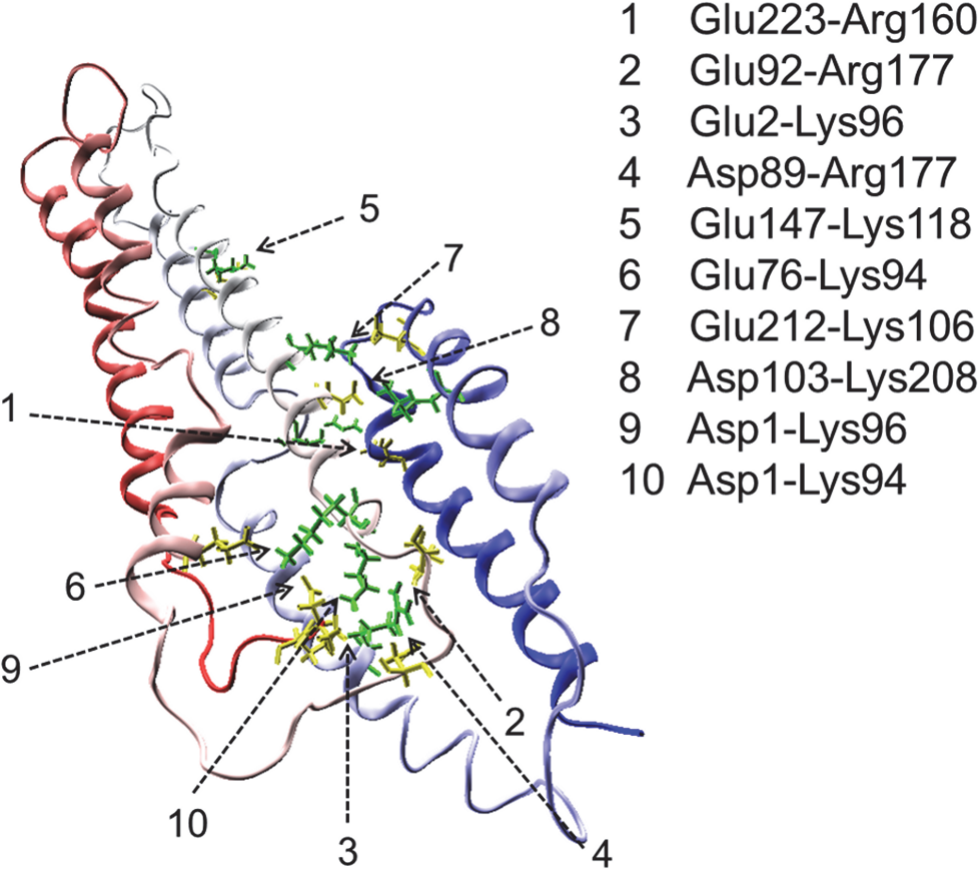
Salt bridge distribution of lipid-free apo A-I MD model. The backbone of apo A-I is presented by ribbons and colored by residue index from red (N-terminus) to blue (C-terminus). Inter-helix salt bridges (listed on the right) are presented with atomic bond. Acidic amino acids are colored by yellow and basic amino acids are colored by green.

The salt bridges restrict the relative positions of helices in lipid-free apo A-I. The salt bridges of the CCL/MS, X-ray and MD models in the lipid-free apo A-I structure during simulation is compared in Table 1. The MD model produced the most salt bridges, about 19 intra-helix salt bridges and 10 inter-helix salt bridges, in the last 3.2 ns of each simulation with 4.1Å cutoff distance. The inter-helix salt bridge provided a continuous constraint on tertiary structure (Fig. 5). The inter-helix salt bridges Glu2-Lys96, Asp1-Lys96, Asp1-Lys94, Glu76-Lys94, Glu92-Arg177 and Asp89-Arg177 constructed a compact salt bridge network, which increased the stability of the N-terminal domain helix bundle. Salt bridges Glu223-Arg160, Glu212-Lys106 and Asp103-Lys208 linked the C-terminal and N-terminal domains, and remained over the entire simulation. These salt bridges cooperated with the hydrophobic effect of hydrophobic surface, stabilizing the helix bundle organization. The X-ray model has 14 intra-helix salt bridges and 3 inter-helix salt bridges, exhibiting an extended helix model.

**Table 1:** Comparison of structural features on lipid-free models in simulation.

Model	Helix Content		CCL Consistency		Salt Bridge			
	(%)		(16 in all)		Intra-Helix		Inter-Helix	
	0 ns	15 ns	0 ns	15 ns	0 ns	15 ns	0 ns	15 ns
**CCL/MS**	51.3	60.1	16	11	10	10	3	5
**X-ray**	84.4	86.1	-	-	15	24	3	3
**MD**	58.8	63.8	16	16	32	19	12	10
**CMD**	53.9	56.4	16	16	34	15	12	6

All structural parameters are measured using VMD. Lys pairs that could satisfy the CCL/MS experiment are measured with a cutoff distance of 20 Å. Salt bridges in the input structure of simulations are measured with cutoff a distance of 4.1 Å (in column “0 ns”). Salt bridges in the output structure of simulations are measured within the last 3.2 ns of each simulation as an averaged distance less than 4.1 Å (in column “15 ns”).

### Structural flexibility in the MD model

**Fig 6:**
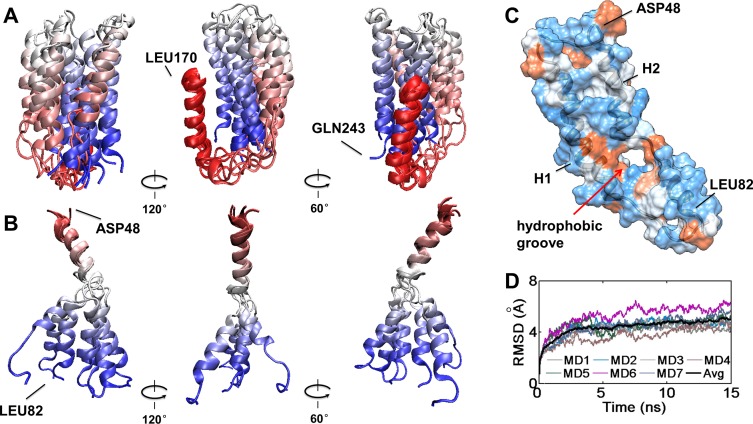
Alternative conformation of local structure in the MD model. A) The variable position of H5 and H6. The backbone of apo A-I C-terminus is presented in ribbons. Structures were aligned by backbone of residue 170 to 185. B) The changeable center angle in H2. The backbone of apo A-I is presented in ribbons. Structures were aligned by the backbone of residues 51 to 62. The ribbon is colored by index from red to blue. C) Surface hydrophobicity representation of MD model (only helix 1 and 2 are displayed). Hydrophobic surface is represented in orange, while hydrophilic surface is represented in blue. D) RMSDs of multiple simulations with the MD model. RMSDs of all protein atoms were measured over the entire trajectory of over seven simulations of the MD model (MD1 to MD7 indicate seven simulations, respectively).

The MD model was most stable throughout the simulation and was most compatible with published experimental data. Seven additional simulations were conducted in order to explore structural features and adjoined stable states. The RMSDs of all seven 15 ns simulations and their averages were plotted in Fig. 6D. The most obvious phenomena in these simulations were the drifting of the C-terminal domain relative to the N-terminal domain and the bending of helix 2.

In multiple simulations, the C-terminal domain drift was observed (Fig. 6A), and may be caused by the weak interactions between the N and C-terminal domains. Comparing conformations (Fig. 1) of the MD and CMD models, the conformational difference in helix 5 does not seriously influence the structure or the flexibility of the N-terminal domain. It has been found experimentally that the C-terminal domain is a relatively isolated part located near the N-terminal helix bundle [50, 59]. Our results show the salt bridges and hydrophobic interactions are the main effect holding the two terminal domains together, which is also consistent with the conclusion that the electrostatic interactions between the C and N-terminal domains are involved in the stabilization of N-terminal helix bundle in the lipid-free apo A-I structure [28, 50].

In these simulations, the central angle of helix 2 exhibited a notable fluctuation (Fig. 6B). Distances between residues 50 to 83 and 50 to 173 have been measured to be near 21∼22Å by FRET [27], which is much smaller than those produced by the CCL/MS model (residues 50 to 83 43.5∼53.5 Å, residues 50 to 173 47.2∼73.5 Å, measured during 15 ns simulation). The alterable central bend in helix 2 of the MD model allows variable distances between residues 50 to 83 (40.7∼56.8 Å) and 50 to 173 (38.1∼52.6 Å), which provides helix 1 and 2 a variable surface. Helix 1 covers the hydrophobic surface of helix 2. This may explain why the amino-terminal deletion mutant studies (removal of residues 1–43) significantly destabilizes the whole lipid-free apo A-I structure [59, 60]. The surface hydrophobicity of the N-terminal region in the lipid-free apo A-I shows a small hydrophobic surface in helices 1 and 2 (Fig. 6C). As the central angle of helix 2 changes, the N-terminal side of apo A-I can gradually form a variable hydrophobic groove with negative curvature wherein lipids may be absorbed and helix-lipid interactions replace helix-helix contacts [61]. This is consistent with previous literature in that the N-terminal residue 44–65 and C-terminal residue 210–241 are reported to make a great contribution in initial lipid association [6]. This may contribute to the lipid binding process of lipid free apo A-I and the formation of preβ HDL.

Our results support the two-step mechanism for lipid binding [28]: in the first step, the C-terminal domain interacts with the lipid surface. Our model shows high mobility in solution which allows the C-terminal domain to change freely for lipid surface searching and activate the binding process. In the second step, the helix bundle opens and forms the globular shape. Our model shows the central part of helix 1 and 2 is flexible and highly hydrophobic, which may allow conformational change through lipid recruitment.

Recently, preβ1 HDL in human blood plasma, as the beginning of RCT process, was reported in a lipid-free state [62]. Our model, through agreement with other preβ1 HDL functions, shows the structure responsible for a critical initial phase of cholesterol uptake.

## Conclusion

A new lipid-free apo A-I MD model, constructed through secondary structure analysis and MD simulations after investigating the stability of X-ray and CCL/MS models, has shown stability under biological conditions during simulations. The first two helices in N-terminal domain (residue 1–80) shows common structural characteristics with the X-ray model, while the LYS distribution of the full structure conformed to CCL/MS experimental measurements. Multiple simulations of the MD model showed structural fluctuations in lipid-free apo A-I, indicating a deformable N-terminal domain and a relatively independent mobile C-terminal domain, whose detailed features were shown to be compatible with experimental knowledge. This MD model, comprised of experimental results from different sources is a possible approach to determine a native structure for lipid-free apo A-I.

## Supporting Information

S1 Datasets The atomic coordinates of MD and CMD model in PDB format. (ZIP)

S1 Fig Construction diversity of the helix bundle in lipid-free apo A-I structure. Two kinds of total shapes were compared to experimental information. Shape one shows better agreement with previous studies. Eight possible configurations of six helices formats are presented. Colored dots represent helices and dark lines represent connecting loops. By detailed simulation tests and comparisons to experimental results, construction shape a2 was selected as a template shape, with final MD model shown on the top right.
(TIF)

S2 Fig Helix hydrophobicity pattern in apo A-I secondary structure. Hydrophobicity pattern of helices in N-terminal domain (residue 1–192) were represented by a colored helix wheel map. The hydrophobic residues are colored green (also presented as diamonds), and green changes into yellow as the hydrophobicity decreases. Hydrophilic residues (uncharged) are colored red (also presented as rectangles) and red decreases proportionally as the hydrophilicity decreases. The charged residues are colored light blue (negatively charged as triangles, and positively charged as pentagons). In the final MD model, the hydrophobicity surface is facing each other, which reduced total energy and kept the structure stable.
(TIF)

S3 Fig Interpeptide cross-links in CCL/MS model and MD model. Distance between Lys pairs which identified by cross-link experiment were measured during the simulation. Nt1 indicates N-terminal residue 1.
(TIF)
