# Supplementary figures and images for: A Model of Lipid-Free Apolipoprotein A-I Revealed by Iterative Molecular Dynamics Simulation
Source: PLoS One. 2015 Mar 20;10(3):e0120233. doi: 10.1371/journal.pone.0120233 (PMC4368682; doi:10.1371/journal.pone.0120233)

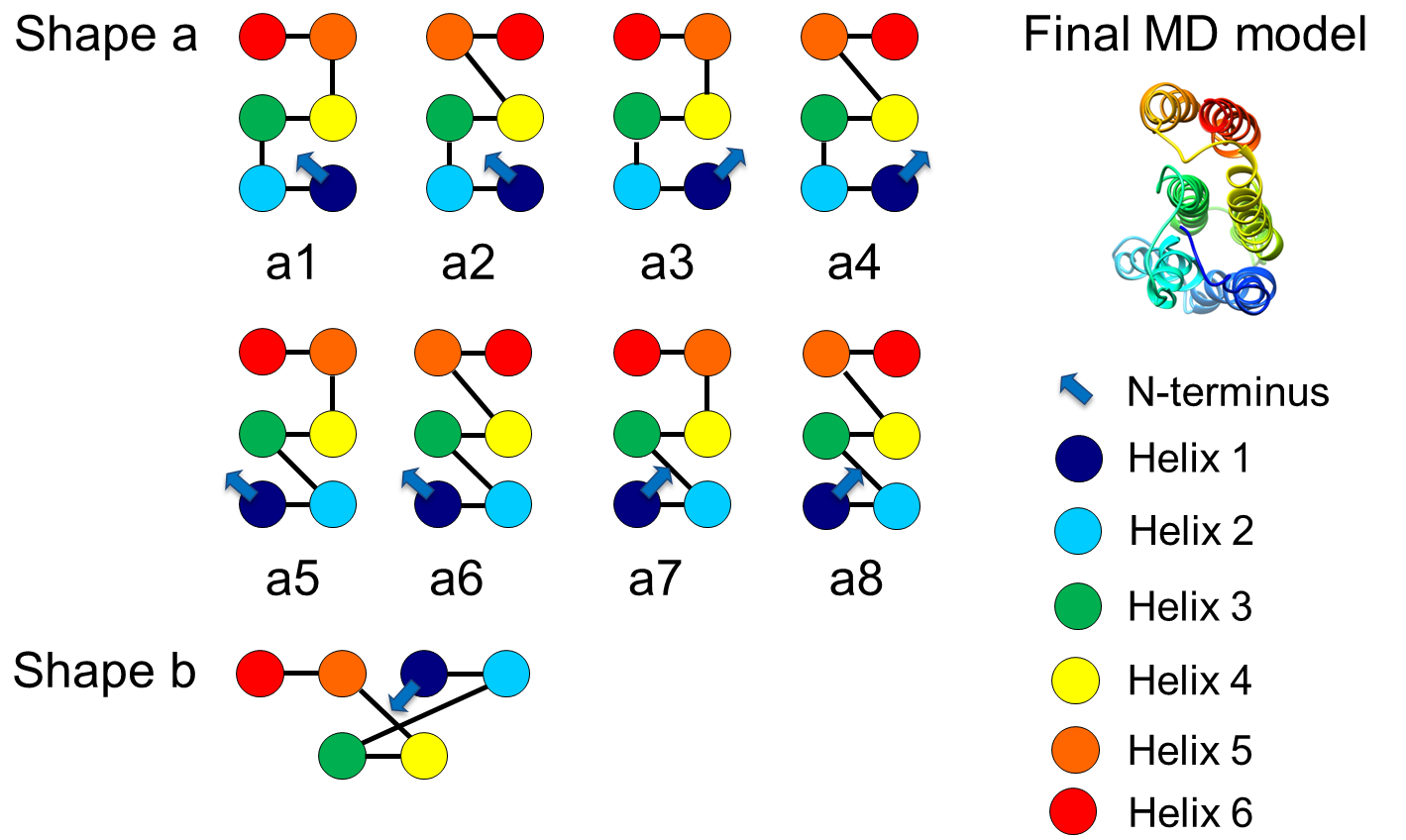

Supplement: S1 Fig — Two kinds of total shapes were compared to experimental information. Shape one shows better agreement with previous studies. Eight possible configurations of six helices formats are presented. Colored dots represent helices and dark lines represent connecting loops. By detailed simulation tests and comparisons to experimental results, construction shape a2 was selected as a template shape, with final MD model shown on the top right. (TIF) [file pone.0120233.s002.tif]

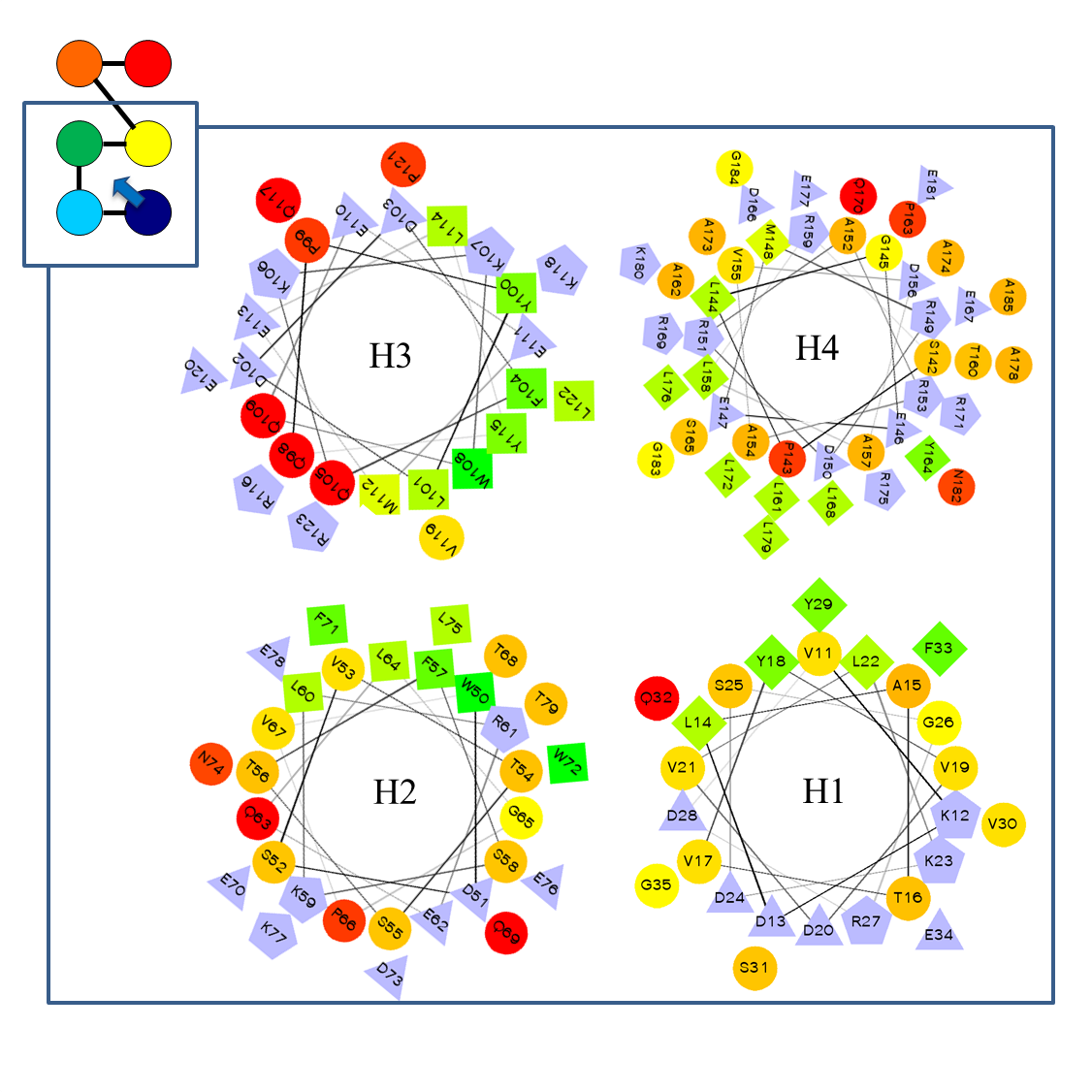

Supplement: S2 Fig — Hydrophobicity pattern of helices in N-terminal domain (residue 1–192) were represented by a colored helix wheel map. The hydrophobic residues are colored green (also presented as diamonds), and green changes into yellow as the hydrophobicity decreases. Hydrophilic residues (uncharged) are colored red (also presented as rectangles) and red decreases proportionally as the hydrophilicity decreases. The charged residues are colored light blue (negatively charged as triangles, and positively charged as pentagons). In the final MD model, the hydrophobicity surface is facing each other, which reduced total energy and kept the structure stable. (TIF) [file pone.0120233.s003.tif]

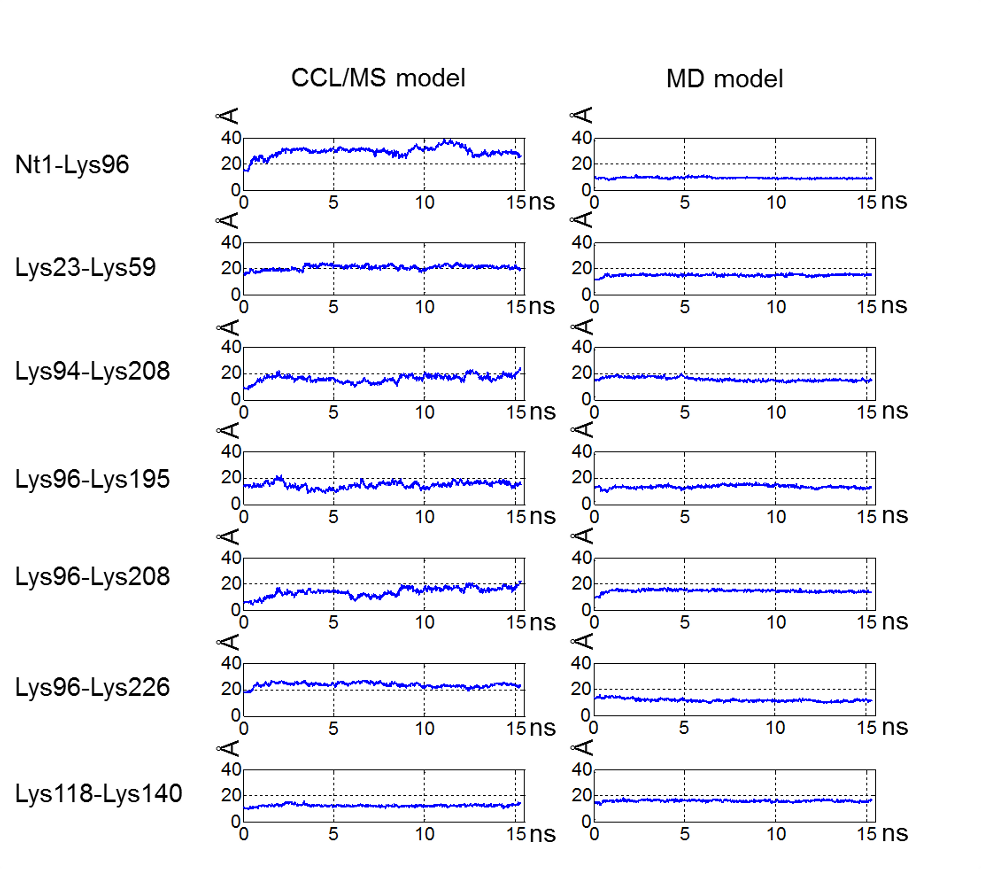

Supplement: S3 Fig — Distance between Lys pairs which identified by cross-link experiment were measured during the simulation. Nt1 indicates N-terminal residue 1. (TIF) [file pone.0120233.s004.tif]
